# Experimental Study on the Phase Transition Characteristics of Asphalt Mixture for Stress Absorbing Membrane Interlayer

**DOI:** 10.3390/ma13020474

**Published:** 2020-01-19

**Authors:** Guang Yang, Xudong Wang, Xingye Zhou, Yanzhu Wang

**Affiliations:** 1 School of Transportation Science and Engineering, Harbin Institute of Technology, Harbin 150090, China; guang.yang@rioh.cn (G.Y.); wangyzh0102@163.com (Y.W.); 2 Research Institute of Highway Ministry of Transport, Beijing 100088, China; zhouxingye1982@163.com

**Keywords:** stress absorbing membrane interlayer, dynamic mechanical analysis, temperature sweep test, phase transition, crumb rubber modified asphalt mixture

## Abstract

Asphalt mixtures used in stress absorbing membrane interlayers (SAMIs) play a significant role in improving the performance of asphalt pavement. To investigate the rheological properties and phase transition characteristics of asphalt mixtures used in SAMI with temperature changes, twenty-seven candidate mixtures with different binders, gradation types and binder contents were selected in this research. During the study, dynamic mechanical analysis method was employed to evaluate their temperature-dependent properties and a series of wide-range temperature sweep tests were conducted under a sinusoidal loading. Some critical points and key indexes from the testing curves such as glass transition temperature (Tg) can be obtained. Test results show that phase transition characteristics can better reflect the rheological properties of asphalt mixtures at different temperatures. Crumb rubber modified asphalt mixtures (AR) provide a better performance at both high and low temperatures. Additionally, the range of AR asphalt mixtures’ effective functioning temperature ΔT is wider, and the slope K value is greater than the others, which indicates that AR asphalt mixtures are less sensitive to temperature changes. Additionally, gradation type and asphalt content also influence the properties: finer gradation and more asphalt content have a good effect on the low-temperature performance of the asphalt mixtures; while mixtures with a coarser gradation and less asphalt content perform better at high temperature and they are less sensitive to temperature changes. Finally, AR asphalt mixture is more suitable to be applied in the SAMI due to its phase transition characteristics from this method.

## 1. Introduction

Stress Absorbing Membrane Interlayer (SAMI) is a thin and soft layer composed of asphalt layers [1], which is widely used as a functional layer between semi-rigid base and asphalt layer or between old asphalt pavement and overlay. Usually, it is used to prevent the reflection cracking and to prevent water entering the base course. Molenaar et al. [2] thought application of a SAMI prevented reflection of cracks through an overlay. Some studies have indicated that the SAMI can delay reflective cracking and plays a role in isolating the overlay from relative deflection of the cracked underlying layer due to traffic loading [3]. For its function of absorbing stress and diffusing deformation, researchers have mainly focused on the mechanical properties of SAMI, such as their shear resistance, flexural-tensile and anti-fatigue characteristics [4,5]. However, their properties with temperature changes have rarely been studied, and extreme weather conditions may lead them to fail quickly.

Asphalt mixtures are typical temperature-sensitive materials and exhibit totally different characteristics at different temperatures. They are usually stiff at low temperature and deform very little under stress, so thermal cracking is induced more easily when a larger thermal stress exceeds their tensile limit; meanwhile, at high temperature, they become more like a fluid, and rutting may occur [6]. With a gradual increase in temperature, the phase of asphalt mixture changes from a glassy state to a highly elastic state, until it finally reaches a viscous state. In engineering applications, asphalt binders are usually modified in order to improve their performance. There are several ways to modify asphalt binders: sulphur, fatty acid amides, polyphosphoric acid, waxes and polymers [7,8]. Furthermore, among polymers, styrene-butadiene-styrene copolymer (SBS), styrene-butadiene rubber (SBR), polyethylene (PE) and ethylene vinyl acetate (EVA) are the most widely used [9,10]. However, the application of modified asphalt binders influences the phase transition process of mixtures, and their rheological characterization requires further study [11]. Phase transition and rheological properties are closely related to pavement distress, so it is very important to evaluate the performance of asphalt mixtures by studying them.

Dynamic Mechanical Analysis (DMA) is an efficient method for studying the rheological properties of viscoelastic materials. Relevant research has demonstrated that the viscoelastic behavior of asphalt binder or cement and asphalt mortar can be studied by this method [12,13]. DMA can be simply described as applying an oscillating force to a sample and analyzing the material’s response to that force. As a typical viscoelastic material, asphalt mixture can be investigated by this method at different temperatures.

From the literature review it can be concluded that recent studies mainly focused on the mechanical properties of asphalt mixtures used in SAMI, such as shear resistance and anti-fatigue performance. However, the effects of environmental factors on the characterization of mixtures have rarely been considered. Investigating the phase transition characteristics of asphalt mixtures with changes in temperature is a more efficient approach to evaluating the high- and low-temperature performance and temperature sensitivity of asphalt mixtures. In this paper, different types of asphalt mixtures are selected to determine which is more suitable for the material in SAMI.

The objective of the present study was to develop a new test method for exploring the rheological and visco-elastic properties of asphalt mixtures used in SAMI with broad temperature changes, and this method can be applied to selecting the optimum asphalt mixture for the SAMI layer. To achieve these objectives, a dynamic mechanical analysis was considered, and temperature sweep tests were conducted on multiple asphalt mixtures with a variety of binder types, gradations and binder contents. High- and low-temperature performance and temperature sensitivity can be compared and evaluated in order to select the optimum mixtures in the SAMI.

## 2. Materials and Methods

### 2.1. Asphalt Binder

Three typical kinds of asphalt were used to study the effect of them on properties of mixtures: neat asphalt binder A70 (70 means the penetration of needle in a binder at 25 °C ranges from 60 to 80 according to the Chinese specification, JTG F40-2004 [14]), SBS modified asphalt, and crumb rubber modified asphalt (AR). They are widely used in the actual production of asphalt mixtures but have obvious differences in properties with temperature and load changing. Table 1 lists the technical index of the three binders.

### 2.2. Asphalt Mixture

#### 2.2.1. Gradation

**Figure 1:**
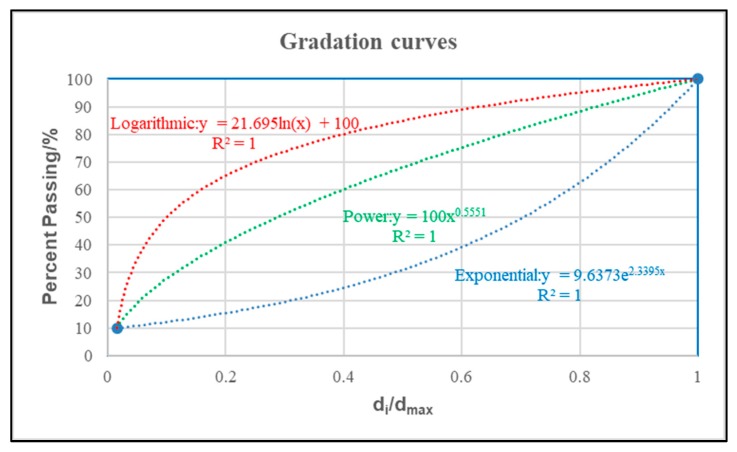
Fitting of gradation curves.

The gradations of asphalt mixtures in this paper were classified by smooth function curves that were fitted by two critical control points. For AC-5 asphalt mixtures (the maximum nominal particle size is less than 5 mm), the passing rate of nominal maximum size and 0.075 mm sieve size were marked as two critical points: their coordinates were (d_max_/d_max_, passing rate at 4.75 mm) and (d_0.075mm_/d_max_, passing rate at 0.075 mm), respectively [15]. Based on the function of asphalt mixtures used for SAMI, the passing rate of 0.075 mm sieve was set as 10%, and that of the nominal maximum size was 100%. As a result, two points (1, 100) and (0.075/4.75 = 0.01579, 10) were calculated. Then, three different functions (logarithmic, power and exponential respectively) were employed to fit the gradation curves, as shown in Figure 1.

**Figure 2:**
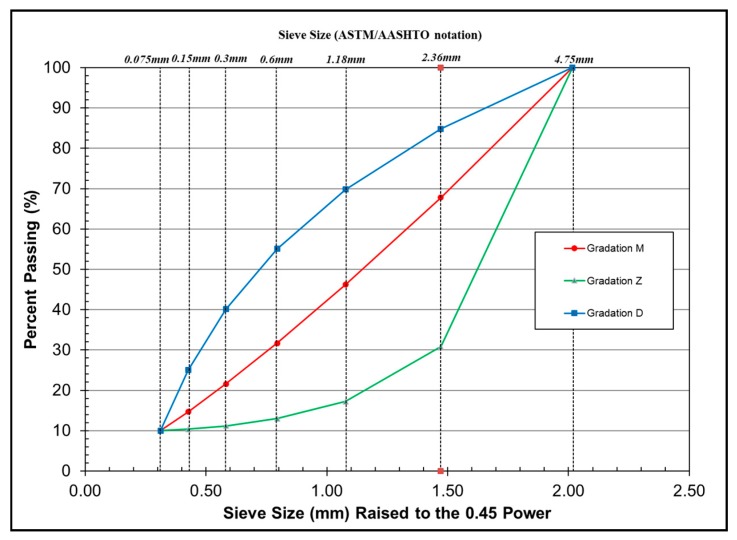
Gradation of AC-5 mixtures.

Finally, three different gradations were determined, marked as M, Z and D. As can be seen from Figure 2, the mixture with gradation D has more fine aggregate, and that with gradation Z is coarser than the other two.

#### 2.2.2. Asphalt Content

Not only the asphalt type and gradation, but also asphalt content directly affects the properties of asphalt mixtures. To make a better comparison of three asphalt mixtures, three asphalt-aggregate ratios were selected by weight for each mixture (5.5%, 6.5% and 7.5%).

**Figure 3:**
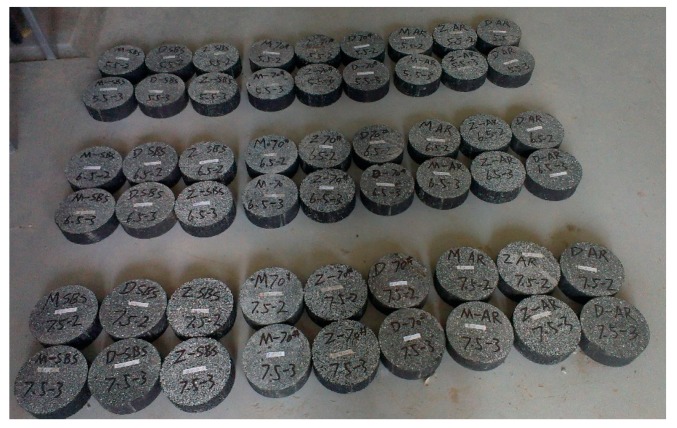
27 AC-5 asphalt mixtures.

To sum up, 27 asphalt mixtures were designed in this test with three asphalt types, three gradations and three asphalt contents, shown in Table 2 and Figure 3.

### 2.3. Experimental Program

#### 2.3.1. Specimen Preparation

The above mixtures were compacted by gyratory compaction method with a diameter of 150 mm. Then both ends were cut to eliminate the uneven distribution of air void. The remaining parts were cut into slices with a high-precision cutting machine (Presi, France). The slices were 60 mm in length, 15 mm in width and 3 mm in thickness. There are six parallel samples for each type of asphalt mixture to ensure the reliability of results.

#### 2.3.2. Experimental Device

**Figure 4:**
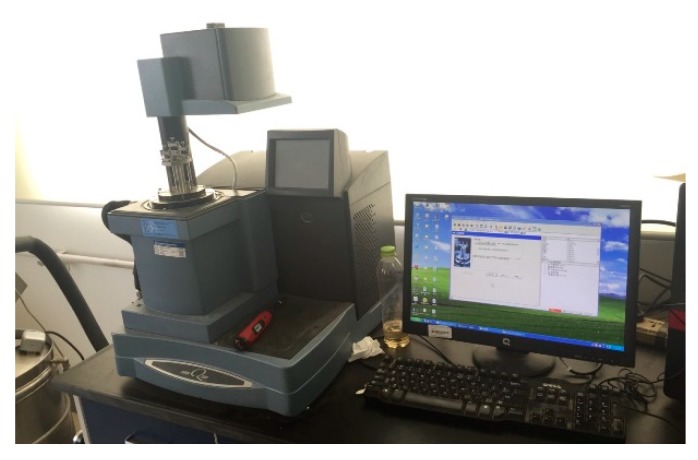
DMA Q800 device.

**Figure 5:**
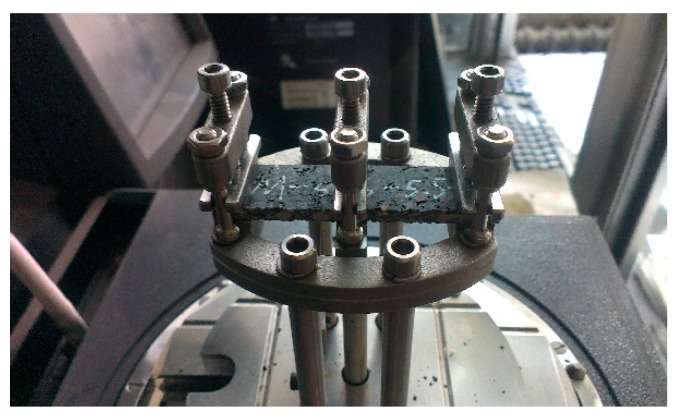
Sample with dual cantilever clamp.

Dynamic Mechanical Analysis (DMA for short) was a practical method to study the rheology properties of viscoelastic materials [16,17]. A Dynamic Mechanical Analyzer called DMA Q800 (TA Instruments, New Castle, DE, USA) was used in this test, shown in Figure 4. It can achieve a wider range of temperature and frequency than other devices for asphalt mixtures and simulate the real environment condition of asphalt pavement at a short time. A dual cantilever clamp was selected to measure the samples under flexural load, shown in Figure 5.

#### 2.3.3. Test Method

The temperature sweep test is an efficient method for studying the materials’ rheology properties in a wide range of temperatures [18,19,20]. The materials were tested under a sinusoidal load with the temperature continuously increasing or decreasing. According to a series of trials, experiments with 1 Hz frequency were chosen as the final parameters. The temperature range is from −40 °C to 80 °C, and the heating rate was set as 2 °C/min, which is more applicable to asphalt mixture.

Before testing, the linear elastic range should be determined for each specimen to ensure elastic deformation within a certain strain level. Finally, 25 με was used as the strain level of temperature sweep test.

#### 2.3.4. Parameter Acquisition

**Figure 6:**
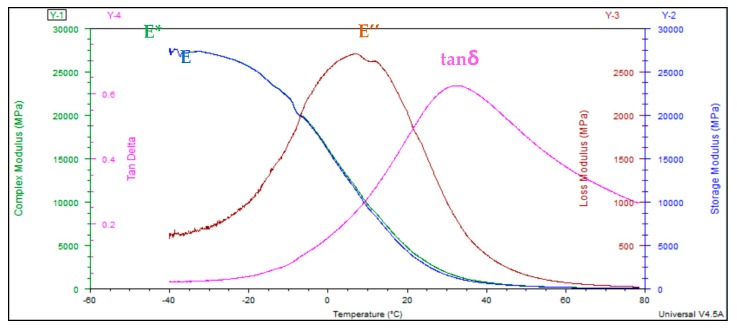
Parameters from temperature sweep test.

From the DMA test, a series of parameters can be obtained by measuring the specimen variation under the sine wave load: Complex modulus E*, storage modulus E′, loss modulus E″ and tangent of phase angle tanδ (see Figure 6). The complex modulus E * is composed of real and imaginary components, referred to as the storage modulus E′ and loss modulus E″. Their correlations can be expressed by the following Equations (1)–(4). The phase transition process from glassy state to highly elastic state and then to viscous state can be obtained by measuring the change in these parameters.

E′ = σ_0_cosδ/ε_0_, (1)

E″ = σ_0_sinδ/ε_0_, (2)

E* = E′ + iE″,(3)

tanδ = E″/E′,(4)

where: σ_0_ is stress amplitude, MPa; ε_0_ is the strain amplitude; δ is the phase angle; E′ is storage modulus, MPa; E″ is loss modulus, MPa; E* is complex modulus, MPa; i = $\sqrt{-1}$.

## 3. Results

**Figure 7:**
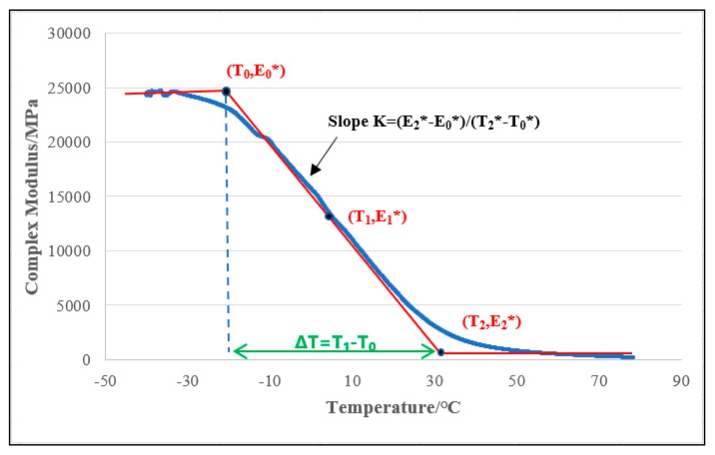
Complex modulus curve.

As can be seen from Figure 7, complex modulus-temperature is a smooth curve, and there exist two platforms at high and low temperatures. With increasing temperature, the complex modulus E* starts to decrease gradually, finally reaching a steady state at high temperature. According to the characteristics of the curve, it can be fitted by the Bolzmann function [21], as shown in Equation (5).

(5) $$y=\frac{A_{1}-A_{2}}{1+e^{\frac{x-x_{0}}{dx}}}+A_{2}$$

where *A*_1_ is the maximum modulus; *A*_2_ is the minimum modulus; *x*_0_ and dx are two parameters that describe the shape of the curve.

Additionally, there was an obvious peak in loss modulus-temperature curve (Figure 6) and the Gauss function can be used to fit the curve and determine the value of the peak, shown in Equation (6).

(6) $$y=y_{0}+\frac{A}{\omega \sqrt{\pi /2}}e^{-2\frac{(x-x_{c}){}^{2}}{\omega^{2}}}$$

where *x_c_* is the temperature where the maximum of loss modulus occurs; *y*_0_ is the minimum of the loss modulus; *A* and ω are two parameters that describe the shape of the curve.

Likewise, there was also a peak in the tangent of phase angle-temperature curve. The GaussAmp function can be used to fit this curve better and estimate the value of the peak, as shown in Equation (7).

(7) $$y=y_{0}+Ae^{-\frac{{\left(x-x_{c}\right)}^{2}}{2\omega^{2}}}$$

where *y*_0_ is the minimum of tanδ; *x_c_* is the temperature where the maximum of tanδ occurs; A and ω are two parameters that describe the shape of the curve.

Based on the above functional fitting, critical points of different curves can be obtained and rheological properties of asphalt mixtures with temperature change can be represented by these points.

### 3.1. Glass Transition Temperature Tg

Glass transition is the reversible transition from a glassy state into a viscous or rubbery state with increasing temperature. Some researchers have stated that Tg is closely related to the low-temperature performance of asphalt mixture [22]. There are several test methods that can obtain Tg, such as DSC, NMR and DMA. Research indicates that the DMA method can measure the asphalt Tg of asphalt-filler mastics accurately by applying a sine wave load on the sample [23]. Lower Tg temperature corresponds to higher fracture energy, which means better low temperature performance of asphalt pavement [24]. Asphalt mixture with lower Tg means it transfers from the high-elastic state to the glassy state at a lower temperature, which indicates that it has a better performance in resisting low-temperature impact. From the DMA method, three critical points can be used as the Tg: onset temperature of E′ curve, temperature at peak of loss modulus and temperature at peak of tanδ.

**Figure 8:**
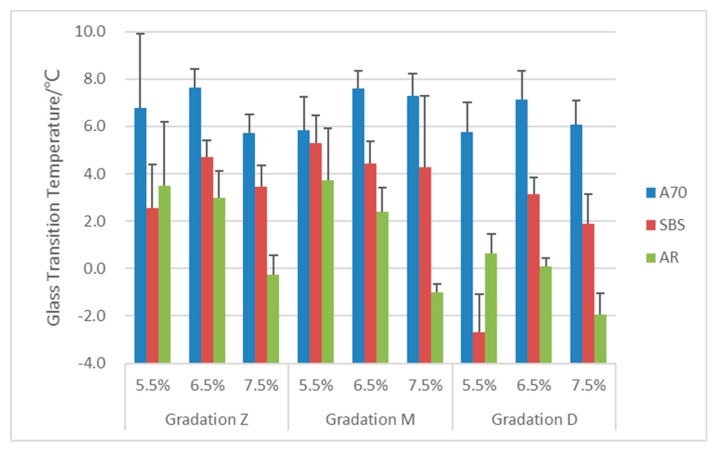
Tg of asphalt mixtures.

The objective of study in this paper was asphalt mixture, which was a mix of asphalt, aggregate and mineral powder. The corresponding temperature at peak of tanδ was usually above 40 °C, and it was in the high-temperature region for asphalt pavement. At the same time, the onset temperature of E′ was not easy to determine, because of the curve shape. Ultimately, the temperature at the peak of the loss modulus was selected as the Tg. The results are shown in Figure 8.

Test results indicate that AR asphalt mixtures have a lower Tg (except SBS mixture with gradation D and 5.5% asphalt content) no matter what the gradation or asphalt content is. It is indicated that AR asphalt has a better low-temperature performance than the other two mixtures. Compared with the other two mixtures, those with neat asphalt A70 have a higher Tg, which means that polymer modifier and crumb rubber have a positive effect on the asphalt mixture to improve their low-temperature performance.

Additionally, gradation type also affected the value of Tg, but the results differed with different asphalt types. For SBS and AR asphalt mixtures, there is a decreasing trend of Tg with the increasing content of fine aggregates from Z to D. In particular, Tg of the finest gradation D decreased obviously than the other two gradations. However, there is little change for Tg of A70 asphalt mixtures with three gradations. It can be concluded from the result that the increasing of fine aggregate content would benefit the low-temperature performance of asphalt mixtures, but it has little effect on the mixtures with neat asphalt.

Moreover, the influence of asphalt content on the Tg was different for the three binder types. For AR asphalt mixtures, Tg decreased a lot with increasing asphalt content, especially at 7.5%. The sufficient asphalt rubber can help mixture release the thermal stress with decreasing temperature. For A70 asphalt mixtures, there exists a peak at 6.5% with a higher Tg, and there was no clear law for SBS asphalt mixtures.

In summary, asphalt type in the mixtures plays a more significant role to the low-temperature performance, followed by gradation type and asphalt content. AR asphalt mixtures have a better low-temperature performance than the other two mixtures.

### 3.2. Stiffness at Extreme Low Temperature E_0_*

**Figure 9:**
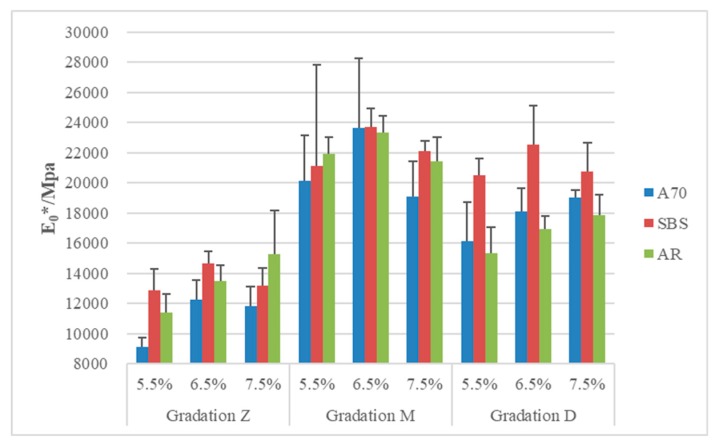
E_0_* of asphalt mixtures (MPa).

The stiffness of asphalt mixtures at low temperature is also an indication of its performance. Some studies have demonstrated that lower stiffness has a better low-temperature performance [18,22]. It can be seen from Figure 7, the complex modulus approaches a constant when the temperature is extreme low for asphalt pavement (usually below −30 °C). From the fitting curve of the complex modulus, the critical point (T_0_, E_0_*) (Figure 7) can be obtained and E_0_* can be used to represent the stiffness of mixture at extreme low temperature, shown in Figure 9.

Based on these results, asphalt mixtures with different asphalt types do not have obvious trends in stiffness at extreme low temperature. For gradation M, the stiffness of asphalt mixtures was almost the same among three asphalt types. Similarly, values of mixtures with different asphalt contents fluctuated within a narrow range. This is due to the condition that the mixtures are in the glassy state.

On the contrary, gradation type played a key role on the stiffness. Mixtures with gradation Z had a lower stiffness mainly due to their high air voids. A large number of holes can be seen on the sample of A70 asphalt mixtures with gradation Z. As a result, asphalt mixtures with gradation Z may not have advantage in resistance to water damage. The stiffness reached a peak at gradation M with increasing compactibility, but higher stiffness means that they cannot make a larger deformation under the same stress. From the gradation M to D, the content of fine aggregates increased further, but the stiffness decreased instead, which is an optimal condition for low-temperature performance of asphalt mixtures.

Factors that influence the stiffness were more complex than those on the Tg. For example, increasing air void may decrease the stiffness of asphalt mixtures such as gradation Z of A70 asphalt, but this was adverse to the low-temperature performance and waterproofness. Lower stiffness with less air void, like gradation D, is more beneficial with respect to resistance to thermal cracking.

### 3.3. High-Temperature Properties T_tan_

**Figure 10:**
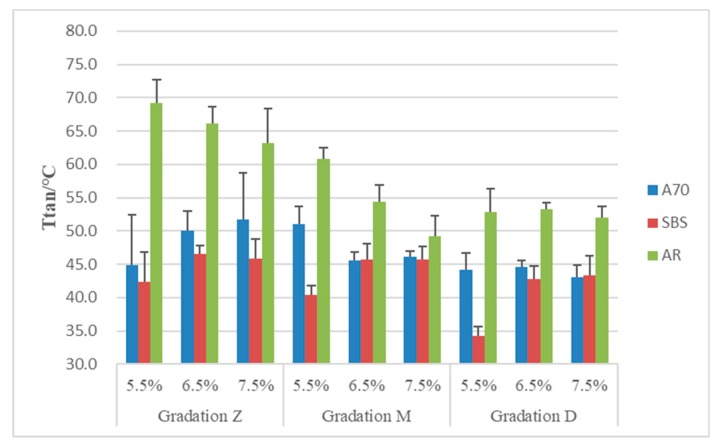
T_tan_ of asphalt mixtures.

As mentioned previously, Tg can be determined by the peak of tangent of phase angle. However, the value of peak was usually above 40 °C for the asphalt mixture, which is in the region of high temperature for asphalt pavement. A higher T_tan_ means it transfers from a highly elastic state to a viscous state at a higher temperature, which is beneficial to high-temperature performance. From the test, it can be observed that after the peak point of tanδ, the sample started to yield, and the stiffness of the asphalt remained at a lower level. Therefore, it can be used to evaluate the high-temperature performance of asphalt mixtures and it has a clear physical meaning. The results of T_tan_ are shown in Figure 10.

The results show that AR asphalt mixtures have an obviously higher T_tan_ than the other two mixtures. It can be found that they performed better at higher temperature. Additionally, gradation Z with coarser aggregates have a higher T_tan_. This is mainly because coarser aggregates interlock with each other and form a skeleton structure to resisting permanent deformation.

### 3.4. Range of Effective Function Temperature ΔT

**Figure 11:**
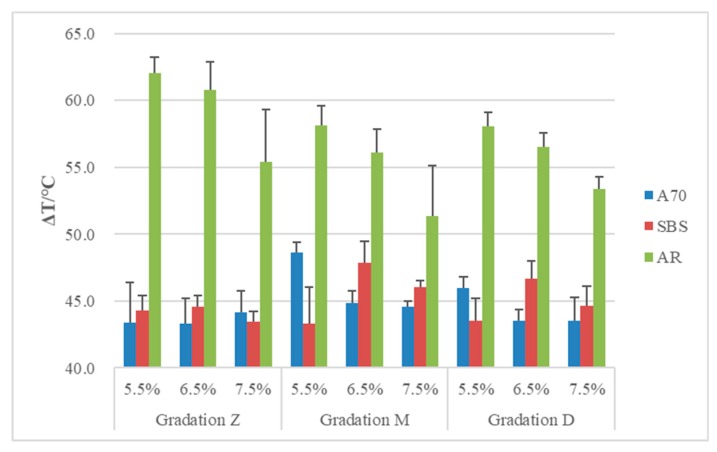
ΔT of asphalt mixtures.

Based on the E* graph, the curve starts to turn with increasing temperature. T_0_ can be used to describe the turning temperature of phase transition. With a gradual increase in temperature, it reaches a platform, and T_2_ can also be marked as the turning temperature for another phase transition. As a result, ΔT = T_2_ − T_0_ shows the range of temperature that can work well for this asphalt mixture, and material in this range was neither too glassy nor too soft. A larger ΔT means it is highly adaptable to temperature change and has a better performance. The results of ΔT are shown in Figure 11.

Results indicate that AR asphalt mixtures have a wider effective functional zone than the other two mixtures. For A70 and SBS asphalt mixtures, the effects of gradation and asphalt content on ΔT are not obvious. For AR asphalt mixtures, there are no obvious differences between different gradation types. However, with increasing asphalt content, ΔT becomes smaller, which means more asphalt makes its range narrower and mixtures are more sensitive to temperature change.

### 3.5. Temperature Sensitivity K Value

**Figure 12:**
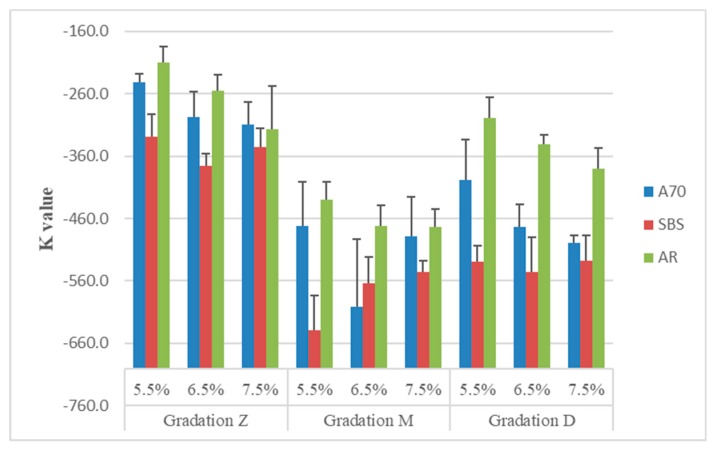
K value of asphalt mixtures.

From the E* curve, the value of modulus decreases from 10^4^ to 10^2^ MPa with the temperature increasing, which means a significant change to asphalt pavement. The slope K of the curve can be used to represent the sensitivity of materials under the effect of temperature. K value can be calculated by Equation (8). The results are shown in Figure 12:

K = (E_2_* − E_0_*)/(T_2_ − T_0_) (8)

The results indicate that asphalt mixtures with gradation Z have a higher K, which means their complex moduli have the minimum change with increasing temperature. One reason is that their lower moduli at low temperature cause lower ΔE, on the other hand, coarser gradation has a better ability to release stress than the other two mixtures.

As for asphalt type, AR asphalt mixtures have a higher K value than the others, which indicates that they have a smaller change of moduli. The K values of A70 mixtures are higher than those of SBS mixtures. The results indicate that SBS asphalt mixtures are most sensitive to temperature changes.

As for binder content, the results show that AR asphalt mixtures containing more asphalt were more sensitive to temperature changes. A70 asphalt mixtures with gradation Z and D have a similar rule. There are no obvious rules for SBS asphalt mixtures.

In terms of gradation type, mixtures of gradation M have a lower K, followed by D and Z. If air void is considered, gradation D is more applicable in SAMI.

In conclusion, the effects of asphalt type, gradation type and binder content on the different indicators can be concluded in Table 3.

The above five indicators can be used to describe phase transition characteristics of asphalt mixtures with temperature changes, and then a comprehensive evaluation of their performance can be made. Tg and E* can characterize the low-temperature properties of mixtures. T_tan_ reflects their stability at higher temperature. ΔT and K value show their temperature sensitivity and effective function range. The test results indicate that the three variables have different effects on the five indicators, and the key impact factor changes with each indicator.

## 4. Conclusions

Based on the testing and analysis, there are some conclusions that can be summarized as follows:

(1) By measuring the glass transition temperature Tg, it can be concluded that crumb rubber modified asphalt mixtures AR with finer gradation D and higher asphalt content have a better low-temperature performance. Asphalt type has the largest impact on the Tg, which shows that asphalt plays a key role in low-temperature properties.
(2) For stiffness at extreme low temperature, values of E_0_* among different asphalt types are similar. The gradation type plays a major role for E_0_* and mixtures with gradation M are larger than the others. Stiffness of mixtures with gradation Z is very low due to its higher air void and it is detrimental to waterproofness. AR asphalt mixtures with gradation D has a relative lower stiffness and it is beneficial to the low-temperature performance.
(3) According to phase transition characterization, T_tan_ (temperature at peak of tanδ) can be used to evaluate the high-temperature performance of asphalt mixtures. Coarser gradation has a good effect on the high-temperature performance. Mixtures with AR asphalt have an obvious higher T_tan_ and they have a better high-temperature stability.
(4) Range of effective function temperature ΔT can be used to describe the range of temperatures where asphalt mixtures can stay in a high-elastic state and work well. Slope K value can be used to evaluate the sensitivity of stiffness to temperature changes. Results show that AR asphalt mixtures have a wider ΔT and a lower K, which indicates that they are less sensitive to various temperatures. Besides, mixtures with more asphalt content and finer gradation are more sensitive to temperature changes.
(5) Considering the comprehensive evaluation of various indicators, AR asphalt mixtures are more suitable materials to be applied in SAMI. An optimum gradation type and asphalt content can be determined from the actual environmental condition and application requirement.

## Figures and Tables

**Table 1:** Technical index of asphalt binder.

Technical Index	A70	SBS	AR
Penetration/0.1 mm (25 °C)	71.5	63.4	37.8
Softening point/°C	48.4	72.7	70.0
Ductility/cm (5 cm/min, 5 °C)	-	28.5	6.7

**Table 2:** Asphalt mixture types.

Asphalt Type	Gradation	Asphalt Content
A70, SBS and AR	M, Z and D	5.5%, 6.5% and 7.5%

**Table 3:** Summary of the results.

Index	Binder Type	Gradation Type	Binder Content
Tg	* AR < SBS < A70	D < M ≈ Z	7.5 < 6.5 < 5.5 (AR)
E_0_*	Not clear	* Z < D < M	Not clear
T_tan_	* AR > A70 ≈ SBS	Z > M > D	Not clear
ΔT	* AR > SBS ≈ A70	Not clear	5.5 > 6.5 > 7.5 (AR)
K value	AR > A70 > SBS	* Z > D > M	5.5 > 6.5 > 7.5 (AR)

* means the key impact factor among three variables.
